# Integrated Transcriptomic and Metabolomic Analysis Reveals Regulatory Effects of Fermented Chinese Chive on Early Testicular Development in Piglets

**DOI:** 10.3390/antiox14091056

**Published:** 2025-08-28

**Authors:** Yupeng Xie, Suthar Teerath Kumar, Hong Zou, Ting-Ting Luo, Yunpeng Zhang, Qi Zhang, Yang Li, Kai-Min Niu, Zhenya Zhai, Chunfeng Wang, Wu-Sheng Sun, Shu-Min Zhang

**Affiliations:** 1 College of Veterinary Medicine, College of Animal Science and Technology, Jilin Provincial Engineering Research Center of Animal Probiotics, Jilin Provincial Key Laboratory of Animal Microecology and Healthy Breeding, Engineering Research Center of Microecological Vaccines (Drugs) for Major Animal Diseases, Ministry of Education, Jilin Agricultural University, Changchun 130118, China; xieyupeng@mails.jlau.edu.cn (Y.X.); zouhong@mails.jlau.edu.cn (H.Z.); luotingting@mails.jlau.edu.cn (T.-T.L.); liyang@mails.jlau.edu.cn (Y.L.); wangchunfeng@jlau.edu.cn (C.W.); 2 College of Animal Science and Technology, Key Laboratory of Animal Production, Product Quality and Security, Ministry of Education, Jilin Agricultural University, Changchun 130118, China; kumar@mails.jlau.edu.cn (S.T.K.); zhangyunpeng@mails.jlau.edu.cn (Y.Z.); zhangqi@mails.jlau.edu.cn (Q.Z.); 3 Institute of Animal Husbandry and Veterinary, Jilin Academy of Agricultural Sciences, Changchun 136100, China; 4 Institute of Biological Resource, Jiangxi Academy of Sciences, Nanchang 330029, China; niukaimin@jxas.ac.cn (K.-M.N.); zhaizhenya@jxas.ac.cn (Z.Z.)

**Keywords:** transcriptomics, metabolomics, fermented Chinese chive, suckling piglets, testis, bioinformatics

## Abstract

Early testicular development is vital for adult male fertility but remains highly vulnerable to stress during the suckling stage. Fermented Chinese chive (*Allium tuberosum*) is known for its antioxidant and immunomodulatory properties, yet its role in testicular development remains unclear. In this study, Songliao Black piglets received 3‰ fermented Chinese chive (LK group) mixed with starter feed and compared to a control (OD group). Testicular samples at weaning (28 days) underwent transcriptomic and metabolomic analyses. Although no significant differences were observed in gross testicular morphology, the LK group significantly increased individual (13.85%) and litter (15.11%) weaning weights (*p* < 0.05), with elevated serum triglycerides, total cholesterol, and a 32.2% rise in IgG levels (*p* < 0.05). Integrated analysis identified 76 shared pathways, including ferroptosis, insulin resistance, PI3K-Akt, MAPK, and cAMP signaling. Upregulated genes in the LK group were mainly related to energy metabolism, antioxidant defense, immune regulation, steroidogenesis, and neuroendocrine signaling, suggesting improved metabolic activity, reduced oxidative stress, and accelerated reproductive maturation. Molecular docking indicated that kaempferol and isorhamnetin from Chinese chive bind strongly to proteins involved in testicular development. Overall, fermented Chinese chive supplementation enhances early testicular development in suckling piglets via integrated modulation of metabolic, immune, and signaling pathways, providing a nutritional strategy to optimize reproductive potential in breeding boars.

## 1. Introduction

Reproductive performance in livestock is shaped by the complex interaction of genetic, nutritional, and environmental factors [1]. Enhancing reproductive efficiency remains a key objective in livestock production, particularly given accumulating evidence of declining semen quality across species exacerbated by environmental pollutants [2]. As the primary site of spermatogenesis and androgen production, early testicular development plays a pivotal role in determining the reproductive capacity of male mammals [3,4]. In males, fertility is primarily determined by semen quality, sperm concentration, and mating capacity, which are closely associated with testicular development and function [5]. This vulnerability is exacerbated by the reproductive system’s heightened susceptibility to environmental contaminants—a sensitivity particularly manifested in sperm’s pronounced response to toxin-induced oxidative stress [6,7]. The suckling period, particularly from birth to weaning, represents a critical developmental phase for testicular maturation in piglets [8]. During this stage, germ cell proliferation, Sertoli cell maturation, and testicular microenvironment establishment collectively determine future spermatogenic capacity [8]. Emerging evidence confirms that environmental pollutants, especially heavy metals like cadmium and arsenic, compromise sperm chromatin architectural proteins, inducing DNA oxidative damage and exerting profoundly adverse effects on reproductive health [9,10,11,12]. Modern intensive farming systems compound these processes with environmental pollutant exposure, weaning stress, and antioxidant deficiencies, thereby synergistically exacerbating oxidative stress and impairing testicular development [13,14,15].

Natural bioactive compounds, particularly flavonoids as a prominent class of phytochemicals, have garnered increasing interest for their regulatory effects on animal development through antagonizing pollutant toxicity via multifaceted mechanisms, including reactive oxygen species (ROS) scavenging, metal chelation, and anti-inflammatory actions [16,17,18]. Allium species, including Chinese chive (*Allium tuberosum*), are rich in polyphenols, organosulfur compounds, and flavonol aglycones (e.g., kaempferol and quercetin) [19,20]. These bioactive constituents confer functional properties as feed additives with demonstrated antimicrobial and antioxidant activities [21]. Microbial fermentation by lactic acid bacteria enhances the flavonoid content of Chinese chive and promotes the biotransformation of polyphenol derivatives, yielding a fermented product with synergistic antioxidant and anti-inflammatory properties [22]. Fermentation can also reduce the pungent odor of Chinese chive, thereby potentially enhancing the palatability of the feed [23]. Our team previously conducted an analysis of the nutritional composition of fermented Chinese chive [22], and fermented Chinese chive was further optimized as a functional feed additive [21]. Fermented products enriched with organic sulfur compounds and flavonoids can specifically mitigate oxidative stress in testicular tissue, potentially supporting reproductive development in mammals. Compared to unfermented sources, these compounds exhibit improved bioavailability and stability, making them more suitable for young animals. Current research on the use of flavonoids from Allium species as feed additives for promoting animal growth, enhancing immunity, and improving antioxidant capacity remains limited [23]. In addition, the underlying molecular mechanisms—particularly those involving gene regulation and metabolic remodeling during key developmental stages—remain poorly understood. This knowledge gap hinders large-scale application due to unclear functional targets and optimal dosing strategies.

Multi-omics integration has emerged as a powerful strategy for deciphering complex traits [24,25]. Transcriptomics enables systematic profiling of transcriptional regulation, screening genetic variants and differentially expressed genes critical for early testicular development [26,27]. Metabolomics reveals post-transcriptional regulatory dynamics through metabolic change detection [28]. Their synergistic application provides deeper mechanistic insights into mammalian testicular development and reproductive capacity. Molecular docking (MD) and dynamics simulations (MDSs) predict small molecule–target protein interactions, elucidating binding affinity and complex stability [29]. The Songliao Black pig is a well-established native breed in China, renowned for its early sexual maturity and strong reproductive performance [30]. The Songliao Black boar, exhibiting early sexual behavior and breeding potential, serves as a suitable model for testicular development studies. This study investigated the effects of fermented Chinese chive on testicular development in suckling piglets using feeding trials integrated with bioinformatics and multi-omics analyses. Key flavonols derived from the fermentation were further evaluated via molecular docking and molecular dynamics simulations to assess their interactions with proteins encoded by genes critical for early testicular development. These findings provide molecular insights into the potential mechanisms by which dietary polyphenols influence mammalian reproductive development.

## 2. Materials and Methods

### 2.1. Ethics Statement

All animal experimental procedures were conducted strictly in accordance with the guidelines established by the Institutional Animal Care and Use Committee (IACUC) of Jilin Agricultural University. The study protocol was approved by the IACUC under approval number (20250703001).

### 2.2. Animal Housing, Sex, and Feeding Management

This study employed 24 healthy primiparous sows with similar expected farrowing dates, randomly allocated to the LK-sow group (n = 12), receiving a basal diet supplemented with 3 g/kg of fermented Chinese chive product, or the OD-sow group (n = 12), receiving a basal diet. One week pre-farrowing, sows were transferred to individual crates where they remained with offspring until weaning. Dietary treatments commenced at crate transfer and continued through lactation (28 days post-farrowing). The piglet treatment group was defined by the maternal group (LK-piglets from LK-sow litters, OD-piglets from OD-sow litters). During 0–7 days postpartum, interventions occurred exclusively via maternal milk. From day 8 to weaning (day 28), piglets received dual-source delivery, via milk from sows and via ad libitum access to starter feed containing an identical concentration of the additive. All progeny from the sows received their respective dietary treatments throughout lactation. At weaning (day 28), 60 male Songliao Black piglets were randomly selected, 30 from LK-sow litters and 30 from OD-sow litters, and designated as the LK group and OD group. The sows and piglets were reared at Gongzhuling National Agricultural Science and Technology, Feimas Animal Husbandry Co., Ltd., Gongzhuling, China. The fermented Chinese chive product was provided by the Institute of Biological Resources, Jiangxi Academy of Sciences (concentration determined based on previous animal trials conducted by the research team). All animals were provided feed ad libitum twice daily at 06:00 and 12:00, with the OD group receiving the basal diet (Table S1) and the LK group receiving the basal diet supplemented with 3 g/kg of fermented Chinese chive product. The preparation process of the fermented Chinese chive was as follows: commercially available Chinese chive were juiced, and then the filtrate was collected and diluted with water at a ratio of 1:4. The diluted chive juice was then fermented at 37 °C for 24 h with Lactobacillus plantarum at an effective viable count of 1 × 10^6^ CFU/mL [21].

### 2.3. Slaughtering Age, Method, and Sample Collection

All experimental piglets were castrated at 28 days of age, and the left testes were collected from each individual. The OD group served as the control, and the LK group represented the treatment group. Collected testes were accurately weighed to determine testis weight. Testicular length, width, and thickness were measured using a vernier caliper (Model: 1012001542; Chengliang Tools, Chengdu, China), and volume was calculated using the formula: V = π/6 × length × width × thickness [31]. Four left testes per group were collected. Portions of each of these testicular samples were fixed in 4% paraformaldehyde for histological examination. The remainder of each tissue sample was immediately snap-frozen in liquid nitrogen and stored at −80 °C for subsequent transcriptomic and metabolomic analyses.

### 2.4. Growth Performance, Serum Biochemical Indices, Serum Antioxidant Indices, and Serum Immune Indices

For suckling piglets, individual birth weight, litter birth weight, survival rate at one week postpartum (number of surviving piglets at one week/number of live-born piglets × 100%), weaning weight at 28 days, and litter weaning weight at 28 days were recorded. Freshly collected blood samples were centrifuged at 3500× *g* for 10 min (4 °C) to separate serum. Separated serum samples were immediately transported on ice packs (0–4 °C) to the Clinical Laboratory of the Affiliated Hospital of Jilin Agricultural University within 2 h, followed by secondary centrifugation at 3000× *g* for 15 min (4 °C). The clarified serum aliquots were stored at −80 °C until analysis. Serum levels of total protein (TP; biuret method), albumin (ALB; bromocresol green), glucose (GLU; glucose oxidase), triglycerides (TG; glycerol phosphate oxidase), and total cholesterol (TC; cholesterol oxidase) were measured using an AU480 Chemistry Analyzer (Beckman Coulter, Brea, CA, USA). Results were reported as g/L (TP and ALB) and mmol/L (GLU, TG, TC). Serum levels of total antioxidant capacity (T-AOC; ABTS method), glutathione peroxidase (GSH-Px; colorimetry), superoxide dismutase (SOD; WST-1 method), and catalase (CAT; visible light) were measured using commercial kits (Nanjing Jiancheng Bioengineering Institute, Nanjing, China; Catalog No. A015-2-1, A005-1-2, A001-3-2, A007-1-1, respectively), following the manufacturer’s protocols. Absorbance was recorded with a BioTek ELX800 microplate reader (Agilent Technologies, Santa Clara, CA, USA), and enzyme activities were calculated as units per milliliter (U/mL). Serum concentrations of immunoglobulin A (IgA), immunoglobulin M (IgM), and immunoglobulin G (IgG) were quantified using porcine-specific ELISA kits (Xiamen Lunchangshuo Biotech Co., Xiamen, China; Catalog No. ED-71134, ED-71143, ED-71136, respectively). The absorbance was measured at 450 nm using a BioTek ELX800 microplate reader (Agilent Technologies, Santa Clara, CA, USA). Results were reported as μg/mL (IgA) and mg/mL (IgM, IgG). All measurements were performed in triplicate to ensure data accuracy and reproducibility, and procedures were followed according to the manufacturer’s guidelines [32].

### 2.5. Preparation of Testicular Tissue Section

The freshly collected testicular tissue was excised and sectioned into approximately 1 cm^3^ blocks. Testicular tissue was firstly fixed in 4% paraformaldehyde at room temperature for over 72 h, then dehydrated in a series of graded ethanol, transferred to xylene, followed by routine paraffin embedding. The paraffin-embedded tissue blocks were trimmed and sectioned to obtain 7 μm thick sections. Hematoxylin and eosin (HE) staining was performed on the sections, which were then examined under a Leica DMI 1 microscope (Wetzlar, Germany). Images of the stained sections were captured and scanned using LAS X (v3.7.5).

### 2.6. Library Construction and Sequencing

Four biological replicates were set up for each group, with each sample subjected to paired-end sequencing in duplicate. Total RNA was extracted from testicular tissue using TRIzol reagent (Invitrogen, Carlsbad, CA, USA), with the concentration and quality assessed by a NanoDrop spectrophotometer (Thermo Fisher Scientific, Waltham, MA, USA). To rigorously evaluate RNA integrity, RNA samples were further analyzed using an Agilent 5400 Bioanalyzer system with the RNA Nano 6000 assay kit (Agilent Technologies Inc., Santa Clara, CA, USA). RNA samples with RNA Integrity Number (RIN) ≥ 8.5 and distinct ribosomal RNA peaks were used for downstream library construction. Approximately 3 μg of RNA was used for library construction: mRNA was purified using oligo(dT) magnetic beads, then fragmented by divalent cations in Illumina proprietary fragmentation buffer. First-strand cDNA was synthesized using random hexamer primers and SuperScript II reverse transcriptase; second-strand cDNA synthesis was performed using DNA Polymerase I and RNase H. The resulting DNA fragments were end-repaired to generate blunt ends via exonuclease/polymerase activities, followed by A-tailing at 3’ ends and ligation of Illumina paired-end (PE) adapters. To select cDNA fragments of 400–500 bp, the libraries were purified using the AMPure XP magnetic bead system (Beckman Coulter, Beverly, CA, USA). Libraries were enriched by 15 cycles of PCR amplification using Illumina PCR primer mix, and PCR products were purified with AMPure XP beads. Library quantification was performed using the Agilent High Sensitivity DNA Kit on a 2100 Bioanalyzer (Agilent Technologies, Santa Clara, CA, USA). The clustering of index-coded samples was performed on a cBot Cluster Generation System using TruSeq PE Cluster Kit v3-cBot-HS (Illumina, San Diego, CA, USA), according to the manufacturer’s instructions. Sequencing was performed on the Illumina NovaSeq 6000 platform by Novogene Co., Ltd. (Beijing, China).

### 2.7. Transcriptomic Data Analysis

Raw FASTQ data were subjected to quality control and filtering using fastp (v0.22.0) to remove low-quality reads [33]. The filtered reads were aligned to the pig reference genome (Sscrofa 11.1) using HISAT2 (v2.1.0) [34]. Gene expression levels were quantified using HTSeq (v0.9.1), and raw read counts were normalized to FPKM (fragments per kilobase of transcript per million mapped reads) [35]. Differentially expressed genes (DEGs) were identified using DESeq (v1.38.3) with the screening criteria of |log2 fold change| > 1 and *p*-value < 0.05 [36]. Bidirectional hierarchical clustering analysis of DEGs was performed using the Pheatmap package (v1.0.12) in R [36]. Gene names were standardized and converted using BioMart [37]. Based on the hypergeometric distribution, Gene Ontology (GO) and KEGG pathway enrichment analyses of DEGs were performed using the clusterProfiler package in R (v3.2.0) [38,39]. Furthermore, Gene Set Enrichment Analysis (GSEA, v4.1.0) was performed to validate the specificity of functional modules [40]. Protein–protein interaction (PPI) networks were constructed using STRING (v12.0) [41]. We constructed the PPI networks analysis of DEGs for the LK and OD groups and used the Maximal Clique Centrality (MCC) method via the cytoHubba plugin in Cytoscape (v3.10.2) to identify hub genes [42].

### 2.8. Metabolite Extraction and Detection

According to the method described by [43], four testicular tissue samples were collected from each group for metabolite analysis. Metabolomic profiling was performed using an ultra-high-performance liquid chromatography system (UHPLC, Thermo Fisher Scientific Vanquish) coupled with an Orbitrap Exploris 120 mass spectrometer (Thermo Fisher Scientific). Chromatographic separation was carried out on a Waters ACQUITY UPLC BEH Amide column (2.1 mm × 50 mm, 1.7 μm particle size). The mobile phase consisted of solvent A (25 mmol/L of ammonium acetate and 25 mmol/L of ammonia solution, pH 9.75) and solvent B (acetonitrile). The autosampler temperature was maintained at 4 °C, and the injection volume was 2 μL. Mass spectrometry data were acquired in information-dependent acquisition (IDA) mode, controlled by Xcalibur software (version 4.3). The electrospray ionization (ESI) source parameters were set as follows: sheath gas flow rate, 50 Arb; auxiliary gas flow rate, 15 Arb; capillary temperature, 320 °C; MS1 resolution, 60,000; MS2 resolution, 15,000; normalized stepped collision energies (NCEs) of 20, 30, and 40; spray voltage of 3.8 kV in positive ion mode and −3.4 kV in negative ion mode.

### 2.9. Metabolite Data Analysis

Non-targeted metabolite analysis was performed based on the total ion chromatogram (TIC). Raw metabolite data acquired under both positive and negative ionization modes were processed using XCMS software (version 4.3) [44]. The raw data were processed to perform peak alignment, retention time correction, and peak area extraction. After data preprocessing, metabolite identification and structural annotation were conducted. Principal component analysis (PCA) was applied to evaluate overall sample distribution and assess the stability of the analytical procedure. Orthogonal partial least squares discriminant analysis (OPLS-DA) and partial least squares discriminant analysis (PLS-DA) were used to distinguish differential metabolites (DMs) among the two groups. The criteria for screening DMs were fold change (|FC|) > 1, variable importance in projection (VIP) > 1, and *p*-value < 0.05. Pearson correlation coefficients were calculated to assess the linear relationships between metabolites. Functional annotation and pathway enrichment analyses were performed based on the KEGG database [45]. Differential metabolites (DMs) were subjected to metabolic pathway enrichment analysis, with pathways considered significantly enriched at *p* < 0.05. Significant pathways were visualized using chord diagrams and bubble plots generated by R software (v3.2.0). Functional Gene Set Enrichment Analysis (GSEA) was performed using the GSEA (v3.0) [46,47].

### 2.10. Integrated Analysis of Transcriptome and Metabolome Data

To clarify the coordinated changes between differentially expressed genes (DEGs) and differential metabolites (DMs) related to spermatogenesis, this study performed an integrative analysis of transcriptomic and metabolomic data. Pearson correlation analysis was then conducted to explore the relationships between commonly annotated metabolites and genes. The correlation between DEGs and DMs was evaluated based on the Pearson correlation coefficient, and the results were visualized through correlation network diagrams for further interpretation.

### 2.11. Molecular Docking and Molecular Dynamics Simulation

We followed the previous study for the analysis of molecular docking and molecular dynamics simulation between genes and metabolites [48]. We obtained the 2D structure of small molecule ligands from the PubChem database (http://pubchem.ncbi.nlm.nih.gov/; accessed on 11 July 2025), and input the 2D structure into ChemOffice (v22.0.0) to generate its 3D structure, saving it as a mol2 file. Then, we used the RCSB PDB database (http://www.rcsb.org/; accessed on 11 July 2025) to select high-resolution crystal structures of protein targets as the receptor for molecular docking. We performed operations such as water removal and dephosphorylation on the protein using PyMOL (v3.1) and saved it as a PDB file. We used Autodock (v1.5.7) to process both the protein and the small molecule structures by adding hydrogens and removing water, and ensuring torsional flexibility of the ligand. Then, define the docking box coordinates. Molecular docking is performed using AutoDock Vina 1.1.2 to explore protein–ligand interactions. The best conformation is determined by comparing the docking scores. The results are visualized using PyMOL and Discovery Studio 2019 Client, and interaction maps between the compound and key residues are analyzed. In addition, molecular dynamics simulation (MDS) is an important tool to analyze the binding affinity and stability of the small molecule–receptor complex. MDS is conducted using Gromacs 2022. Force field parameters are obtained using the pdb2gmx tool in Gromacs and the AutoFF web server. During the simulation, the CHARMM 36 force field is used for the receptor protein [49], and the CGenff force field is applied to the ligand. The system is solvated by adding a 1 nm cubic TIP3P water box around it [50]. The gmx genion tool is used to add ions to achieve electrostatic neutrality. Long-range electrostatic interactions are handled by the Particle Mesh Ewald (PME) method, with a cutoff distance set to 1 nm. All bond constraints are implemented using the SHAKE algorithm, and the Verlet leapfrog algorithm is employed to integrate the molecular dynamics process with a time step of 1 fs. Prior to the molecular dynamics simulation, the system undergoes energy minimization. The energy minimization process includes 3000 steps of steepest descent optimization, followed by 2000 steps of conjugate gradient optimization. The optimization steps are as follows: first, the solute is constrained and the water molecules are minimized; then, the counter ions are constrained and minimized; finally, the entire system is minimized without any constraints. The simulation is run under NPT conditions at 310 K and constant pressure, for a simulation time of 100 ns. During the simulation, the tools g-rmsd, g-rmsf, g-hbond, and g-Rg are used to calculate the root mean square deviation (RMSD), root mean square fluctuation (RMSF), hydrogen bonds (HBonds), radius of gyration (Rg), and Gibbs free energy landscape.

### 2.12. Validation of DEGs

To verify the reliability of the RNA-seq data, six random genes (*IGF-1*, *SAT1*, *GPER1*, *PPP1R3C*, *ADCY13*, and *P2RY13*) were selected for RT-qPCR validation. Each group included four biological replicates, with three technical replicates performed independently per sample. The total RNA used for RT-qPCR was extracted from the same batch as that used for RNA-seq. Primer sequences were designed using NCBI Primer-BLAST and Primer 5.0 software. PCR reaction conditions and system proportions followed protocols from previous studies by our research group, with a final primer concentration of 10 μmol/μL. *GAPDH* was used as the internal reference gene. Statistical analysis of gene expression differences between groups was performed using GraphPad Prism 10, and results are presented as mean ± standard error. Primer nucleotide sequences are listed in Table S2.

### 2.13. Statistical Analysis

All data were analyzed using GraphPad Prism 10, and results are expressed as mean ± standard deviation (mean ± SD). Student’s *t*-test was used to assess the differences between groups, with a significance threshold set at *p* ≤ 0.05. Correlation analyses were conducted and visualized using RStudio (v4.4.2) (Hmisc and pheatmap).

## 3. Results

### 3.1. Histological Observation of Testis

**Figure 1:**
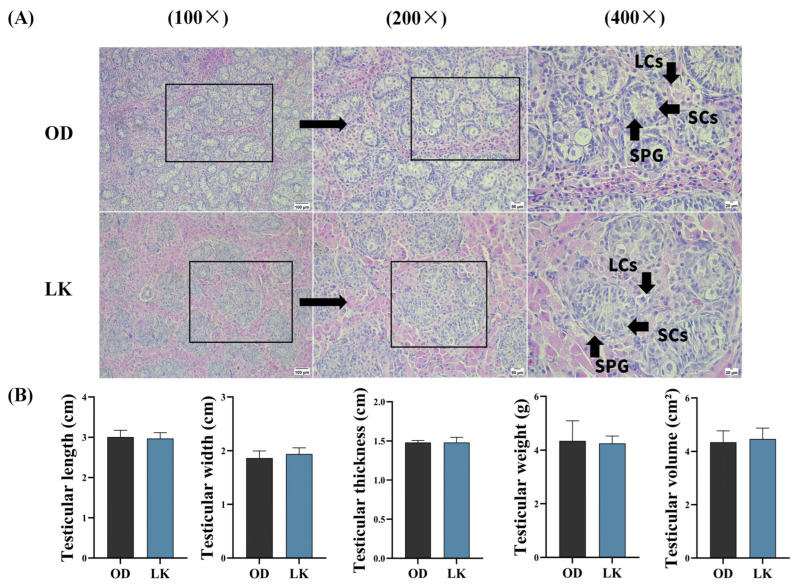
Comparative analysis of hematoxylin and testicular phenotypical parameters between LK and OD groups. (**A**) shows hematoxylin and eosin (HE)-stained sections of testicular tissue from Songliao Black piglets. The first column presents the testicular morphology at 100× magnification with a scale bar of 100 μm. The second column illustrates the tissue morphology at different developmental stages under 200× magnification (scale bar = 50 μm). The third column displays detailed histological features observed at 400× magnification (scale bar = 20 μm). Key cell types identified include spermatogonia (SPG), Sertoli cells (SCs), and Leydig cells (LCs). (**B**) provides the quantitative measurements of the testis, including length, width, thickness, weight, and volume.

Testicular tissue morphology of the OD and LK groups was examined by hematoxylin and eosin (HE) staining (Figure 1A). The results showed that the testicular tissue of suckling piglets was morphologically intact, with seminiferous tubules arranged loosely in a radial pattern. The tubule lumens were narrowed, the seminiferous epithelium was mainly composed of primordial germ cells and a small number of spermatogonia (SPGs) near the basement membrane; whereas spermatocytes and spermatids were absent, columnar Sertoli cells (SCs) were tightly arranged along the inner side of the basement membrane. The testicular interstitium consisted of loose connective tissue, rich in blood vessels and lymphatics, with large interstitial Leydig cells (LCs) exhibiting eosinophilic cytoplasm, indicating active endocrine function. No significant differences in testicular morphology or cellular composition were observed between the OD and LK groups, suggesting that at this stage, testicular development in piglets remains in the early arrest phase of spermatogenesis. Testicular length, width, thickness, weight, and volume showed no significant differences between the OD and LK groups (Figure 1B).

### 3.2. Growth Performance

As shown in Table 1, no significant difference was observed in the piglet survival rate during the first week after birth between the LK and OD groups (*p* > 0.05). However, by day 28, piglets exhibited a 13.85% increase in weaning weight (*p* < 0.05), and the litter weaning weight was 15.11% higher in the LK group compared to the OD group (*p* < 0.05).

### 3.3. Serum Biochemical Indices

As indicated in Table 2, serum biochemical indices in piglets were altered by supplementation. For TP, ALB, and GLUC, concentrations did not differ significantly between the LK and OD groups (*p* > 0.05). However, piglets showed significant increases in TG and TC levels, by 88.18% (*p* < 0.05) and 42.58% (*p* < 0.05), respectively, in the LK group compared to the OD group.

### 3.4. Serum Antioxidant Indices

According to Table 3, serum antioxidant indices in piglets did not differ significantly between LK and OD groups (*p* > 0.05).

### 3.5. Serum Immune Indices

As demonstrated in Table 4, serum immune indices in piglets were altered by supplementation. For IgA and IgM, no significant differences were observed between LK and OD groups (*p* > 0.05). However, piglets showed significant increases in serum IgG levels, by 32.2% (*p* < 0.05), in the LK group compared to the OD group.

### 3.6. Transcriptomic Profiling of Testis

#### 3.6.1. Summary of RNA-Seq Analysis

RNA-seq data from eight samples mapped between 45 and 58 million raw reads per sample. After quality control and alignment, a high percentage of reads successfully mapped to the reference genome, with overall mapping rates ranging from 91.4 to 92.9%. Among these, unique mapping rates ranged from 89.1 to 90.7%, while multi-mapped reads accounted for approximately 2.1–2.3% of total reads. Both read1 and read2 mapping rates were balanced, and strand-specific mapping showed nearly equal distribution between the positive and negative strands. Additionally, more than 84% of reads were mapped in proper pairs, indicating high library quality. Spliced and unspliced reads were also recorded, with spliced reads accounting for 34–37% and unspliced reads accounting for 52–56%, reflecting active transcription and RNA processing (Table S3).

#### 3.6.2. Comparison of RNA Seq Data

**Figure 2:**
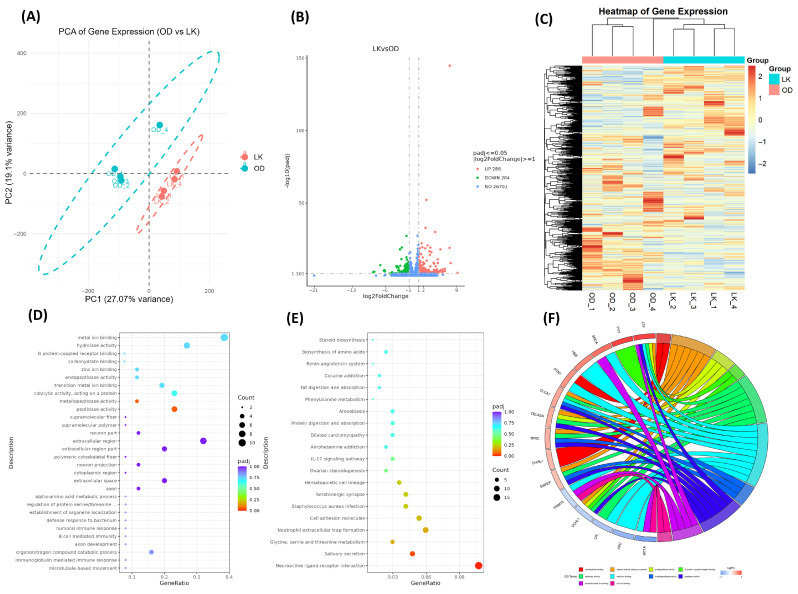
Comparative transcriptomic and functional analysis of DEGs. (**A**) Principal component analysis (PCA) of expressed genes for each sample of the LK and OD groups. (**B**) Volcano plot of significantly differentially expressed genes (DEGs); red dots represent significantly upregulated genes, green dots represent significantly downregulated genes, and blue dots indicate genes without significant differential expression. (**C**) Heatmap clustering analysis of expressed genes within each sample of the LK and OD groups. (**D**) Gene Ontology (GO) enrichment pathway analysis of DEGs. (**E**) Kyoto Encyclopedia of Genes and Genomes (KEGG) functional analysis of DEGs. (**F**) GO enrichment top 10 chord diagram. The right side represents the classification composition, and the left side represents the classification and gene correspondence.

A total of 13816 genes were mapped with the reference genome; among them, 13087 were common genes, while 363 were non-overlapped unique genes in the LK group and 366 in the OD group (Table S4). Along with this, we found 396 novel ids. Principal component analysis (PCA) demonstrated a clear variation in samples from both groups (Figure 2A). Further, we found 570 differentially expressed genes (DEGs), with 284 genes significantly downregulated and 286 genes upregulated (Figure 2B). Hierarchical clustering heatmap analysis further highlighted gene expression differences among samples, with the eight samples from the LK and OD groups clearly differentiated into two distinct clusters (Figure 2C). These findings robustly confirm the presence of significant gene expression differences between feeding groups and underscore the profound regulatory impact of fermentation Chinese chive supplementation on gene expression.

#### 3.6.3. Functional Analysis of DEGs

Differentially expressed genes (DEGs) between the LK and OD groups were analyzed using KEGG and GO pathway enrichment. Several KEGG pathways were identified in the testicular response to fermented Chinese chive supplementation (LK) (Figure 2E; Table S5); enriched neuroactive ligand–receptor interaction contains *PTGER2*, *VIPR2*, *GRPR*, and *CHRNA6* genes, which are crucial for neurotransmitter and hormonal signaling in follicular development, ovulation, and oocyte maturation. The salivary secretion pathway, involving *ADCY2* and *CD38* genes, expresses its importance in cAMP and calcium signaling, essential for granulosa cell function and steroid hormone production. The testicular steroidogenesis pathway included *IGF1*, *ADCY2*, and *HSD17B2*, suggesting a regulatory role in steroid biosynthesis. Immune-related pathways like neutrophil extracellular trap formation and IL-17 signaling point to an immunomodulatory effect, potentially influencing follicular remodeling and ovulation. Pathways related to cell adhesion and serotonergic synapses suggest changes in intercellular communication and neuroendocrine regulation.

GO analysis revealed biological processes (BPs) such as microtubule-based movement and axon development, involving *UCHL1* and *NEFL*, suggesting the regulation of cytoskeletal dynamics and neuronal signaling. Immune-related processes like immunoglobulin-mediated immune response and B cell-mediated immunity, driven by *C7* and *TFRC*, indicated enhanced humoral immune activity. Metabolic adjustments were reflected in the organonitrogen compound catabolic process, involving *TDH*, *HPD*, and *CELA2A*, suggesting changes in amino acid and protein turnover. In the cellular component category, “axon” was prominent, supporting potential neurodevelopmental changes. The molecular function category highlighted peptidase and metallopeptidase activities, with genes such as *ENPEP*, *TFRC*, *CELA2A*, and *CLCA1*, indicating increased proteolytic activity linked to tissue remodeling or immune defense (Figure 2D,F; Table S6). These findings suggest that LK supplementation enhances testicular development by modulating hormonal signaling, immune response, steroidogenesis, and metabolic processes.

#### 3.6.4. Identification of Hub Genes Between LK and OD Groups

**Figure 3:**
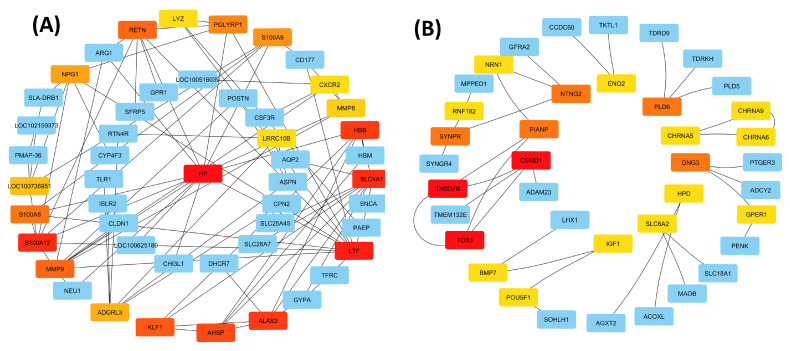
Identification of hub genes from DEGs. (**A**) Protein–protein interaction (PPI) network analysis of upregulated genes in the LK group; from highly red to yellow–blue color, gene nodes are showing a higher interconnectivity score as a strong hub gene. (**B**) Protein–protein interaction (PPI) network analysis of downregulated genes in the OD group; from highly red to yellow–blue color, gene nodes are showing a higher interconnectivity score as a strong hub gene.

We identified the top 20 hub genes from upregulated DEGs expressed in the LK group. Among them, *HP*, *LTF*, *SLC4A1*, *S100A12*, *ALAS2*, *HBB*, *AHSP*, *KLF1*, *MMP9*, *RETN*, *S100A8*, and *PGLYRP1* scored highest. These genes are mainly involved in the immune response, erythropoiesis, and inflammation, suggesting LK treatment activates host defense, red blood cell function, and extracellular matrix remodeling pathways (Figure 3A). In contrast, 20 hub genes were downregulated in the OD group, with *THSD7B*, *TOX3*, and *CSMD1* having the highest scores. These genes are linked to neuronal development, synaptic signaling, and neurotransmission, indicating reduced neuro-regulatory and developmental activity in the OD group (Figure 3B). The contrasting hub gene expression patterns suggest that LK treatment enhances immune and hematopoietic responses, while OD control suppresses neurogenesis-related pathways, offering insights into the molecular effects of each treatment.

### 3.7. Untargeted Metabolomics Sequencing of Testicular Tissue

#### 3.7.1. Classification of Metabolites

A total of 868 negatively ionized metabolites were annotated using the Human Metabolome Database (HMDB), classified into 14 chemical categories. The most abundant were lipids and lipid-like molecules (278), followed by organic acids and derivatives (184) and organoheterocyclic compounds (115). Other notable classes included benzenoids (81), organic oxygen compounds (75), and nucleosides, nucleotides, and analogs (41). Less represented categories included phenylpropanoids and polyketides (24), alkaloids (9), and organohalogen compounds (6), with minimal contributions from organosulfur compounds (5), organic nitrogen compounds (3), lignans (1), and hydrocarbons (1). This distribution reflects a metabolome rich in lipid and organic acid compounds under negative ion mode. In positive ion mode, 1388 metabolites were annotated across 15 chemical categories. Lipids and lipid-like molecules remained the largest group (401), followed by organic acids and derivatives (317) and organoheterocyclic compounds (225). Other prominent classes included benzenoids (94), organic oxygen compounds (74), and phenylpropanoids and polyketides (42). Smaller groups included organic nitrogen compounds (35), nucleosides and analogs (32), and alkaloids (20). Minor classes were organosulfur compounds (6), organohalogens (1), organic 1,3-dipolar compounds (1), lignans (1), and hydrocarbons (1). These findings highlight the chemical diversity of the metabolome, with a strong enrichment of lipid and acid-related metabolites, consistent with ionization efficiencies in both modes.

#### 3.7.2. DEMs Analysis of Testicular Tissues

**Figure 4:**
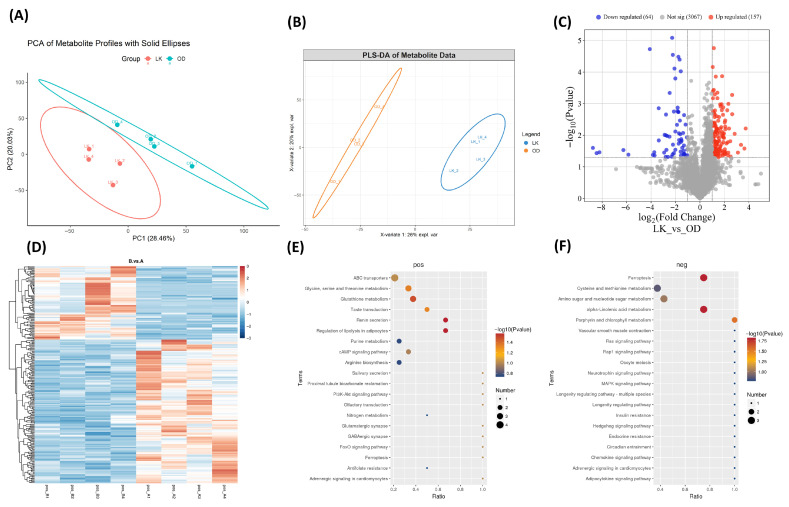
Comparative analysis of testicular metabolites and functional analysis. (**A**) Principal component analysis (PCA) expressed the metabolites of testicular tissue between the LK and OD groups. (**B**) PLS-DA analysis of expressed metabolites. (**C**) Volcano graph of expressed upregulated and downregulated metabolites. (**D**) Heatmap of expressed metabolites. (**E**) Functional KEGG enrichment pathway analysis of positive ion mode. (**F**) Functional KEGG enrichment pathway analysis of negative ion mode.

Non-targeted metabolomic analysis of testicular tissue samples was performed using UHPLC-MS/MS identified a total of 3289 metabolites, among them 1351 were negative and 1938 pos ion charged metabolites (Table S7). PCA and PLS-DA showed notable variation between samples of LK and OD groups (Figure 4A,B). Further, based on |long 2FC| > 1 and a *p*-value < 0.05, we identified 177 upregulated genes in the LK group and 64 downregulated genes in the OD group (Figure 4C). Distribution of DEG metabolites in with samples of both groups is shown in Figure 4D.

#### 3.7.3. KEGG Enrichment Analysis of Differential Metabolites

Functional analyses of differentially expressed metabolites were analyzed through KEGG pathway enrichment for both positive and negative ion mode metabolites. DEMs of positive ion mode were mainly enriched in glutathione metabolism, glycine, serine, and threonine metabolism, and the PI3K-Akt signaling pathway, along with purine metabolism, ABC transporters, and ferroptosis. These findings imply a coordinated regulation of oxidative stress response, nucleotide biosynthesis, and transmembrane substance clearance. Moreover, the FoxO signaling pathway and adrenergic signaling in cardiomyocytes suggest that the integration of cell cycle regulation and local hemodynamics may play an essential role in early testicular development (Figure 4E; Table S8). While negative ion mode DEMs were predominantly enriched in alpha-linolenic acid metabolism, MAPK/Ras/Rap1 signaling pathways, and ferroptosis, indicating a key role for lipid homeostasis and oxidative stress defense in the remodeling of testicular tissue, the hedgehog signaling pathway and endocrine resistance suggest a coordinated regulatory mechanism involving gonadal differentiation and hormonal signaling homeostasis. Additionally, the enrichment of amino sugar and nucleotide sugar metabolism and circadian entrainment points to potential roles of glycosylation and temporal regulation in the interaction between germ cells and supporting cells during reproductive development (Figure 4F; Table S8).

### 3.8. Combined Analysis of Transcriptome and Metabolome of Testicular Tissue

**Figure 5:**
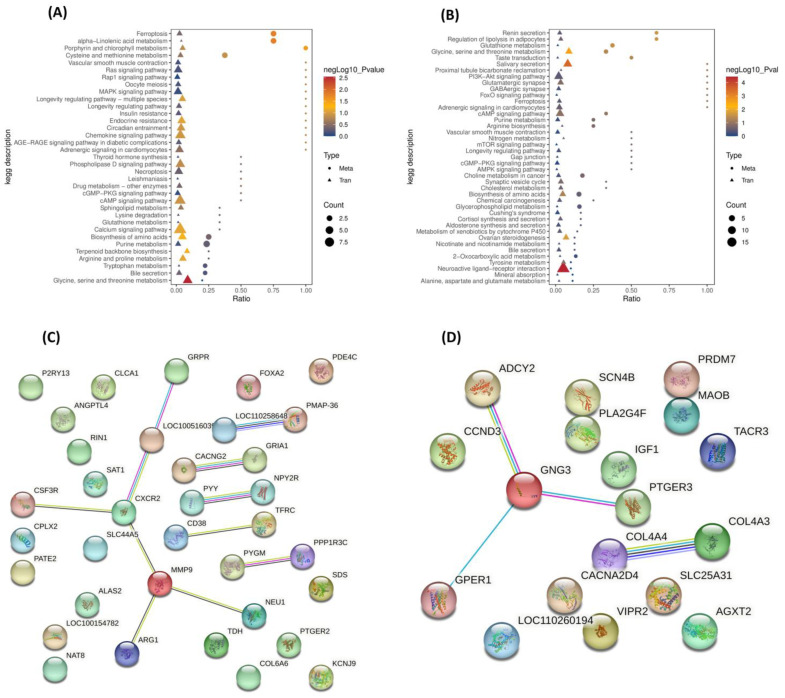
Joint analysis of transcriptome and metabolome. (**A**) Joint analysis of negative ion mode DEMs and DEGs expressed in similar pathways. (**B**) Joint analysis of positive ion mode DEMs and DEGs expressed in similar pathways. (**C**) Protein–protein interaction (PPI) analysis of upregulated DEGs expressed in similar KEGG pathways of transcriptome and metabolome. (**D**) Protein–protein interaction (PPI) analysis of downregulated DEGs expressed in similar KEGG pathways of transcriptome and metabolome.

Joint analysis of DEGs and metabolites revealed substantial overlap in enriched pathways, with 35 shared pathways identified for negative ion metabolites (Figure 5A; Table S9) and 41 for positive ion metabolites (Figure 5B; Table S9). The string-based protein–protein interaction (PPI) network plot for upregulated genes in the LK group and downregulated genes in the OD group is shown in (Figure 5C,D), respectively. Key common pathways included ferroptosis, neuroactive ligand–receptor interaction, insulin resistance, MAPK, Ras, Rap1, cGMP-PKG, and cAMP signaling, showing a total of 58 DEGs between fermented Chinese chive (LK) supplemented and control (OD), with 39 genes upregulated in LK and 19 genes upregulated in OD. Genes elevated in LK were primarily linked to metabolic regulation (*PPP1R3C*, *TDH*, *PYGM, ARG1*, *ANGPTL4*), immune response (*CD38*, *CXCR2*, *CSF3R*, *PYY*), oxidative stress resistance (*TFRC*, *PMAP-36*), cellular signaling and testis structure (*COL6A6*, *FOXA2*, *SAT1*, *RIN1*, *GRIA1*, *KCNJ9*), and neurotransmission (*NPY2R*, *P2RY13*, *GRPR*, *CPLX2*). Conversely, OD-upregulated genes were associated with inflammation and prostaglandin signaling (*PLA2G4F*, *PTGER3*), steroidogenesis and hormone signaling (*IGF1*, *GPER1*, *ADCY2*, *TACR3*), extracellular matrix organization (*COL4A3*, *COL4A4*), and oxidative metabolism (*MAOB*, *AGXT2*). The predominance and diversity of LK-upregulated genes suggest enhanced testicular metabolism, immune function, and structural integrity due to fermented Chinese chive supplementation. Metabolomic analysis highlighted recurring metabolites across multiple pathways: glutathione and γ-glutamylcysteine were prominent in oxidative stress and redox-related pathways, including ferroptosis and cysteine/methionine metabolism. AMP and L-noradrenaline appeared frequently in pathways regulating energy metabolism and neuroendocrine signaling, such as lipolysis, renin secretion, cAMP, PI3K-Akt, FoxO, and adrenergic signaling. L-glutamine was also consistently enriched in amino acid metabolism and neurotransmission pathways like glutamatergic and GABAergic synapses. Additional metabolites like 5-oxoproline and stearidonic acid indicated active redox cycling and omega-3 fatty acid metabolism. These data collectively underscore coordinated metabolic remodeling involving oxidative defense, energy regulation, and lipid signaling in testicular tissue.

### 3.9. Gene–Metabolite Correlation Analysis in Testicular Tissues

**Figure 6:**
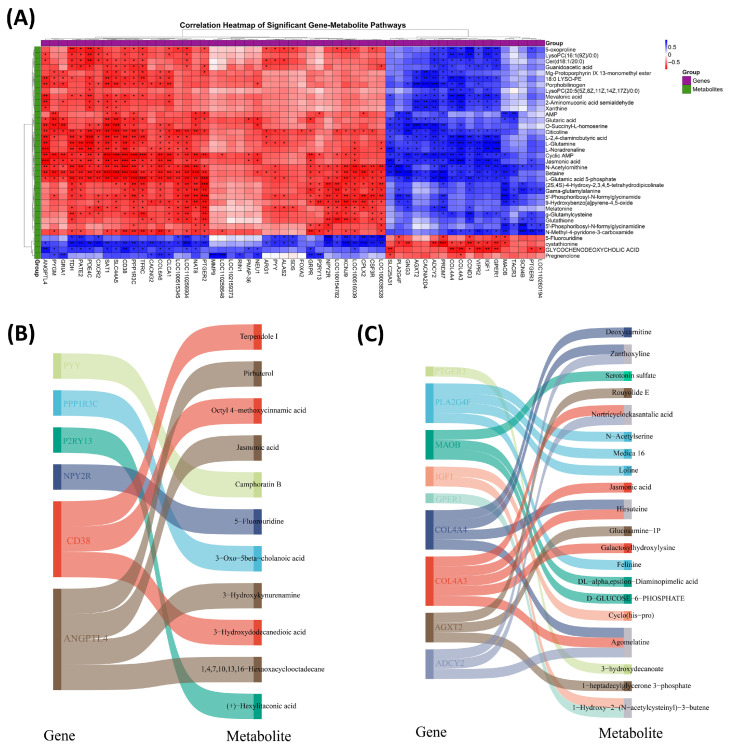
Joint correlation analysis of genes and metabolites. (**A**) Heatmap of gene and metabolite correlations showing the relationship between genes and metabolites in the KEGG pathway significantly enriched by combined transcriptomic and metabolomic analyses of the LK and OD groups. The clustered heatmap visualizes the strength of the correlations and the direction of regulation. Blue color indicates positive correlations and red shows negative correlations. Note: *p* < 0.05 with *, *p* < 0.01 with **, and *p* < 0.001 with *** highlighting the significant level between key genes and metabolites. (**B**) Sankey graph between upregulated genes and metabolites with a correlation r > 0.97. (**C**) Sankey graph between down-regulated genes and metabolites with a correlation r > 0.97.

An integrated Pearson correlation analysis of transcriptomic and metabolomic data from testicular tissues of the LK and OD groups revealed significant gene–metabolite associations (Figure 6A), suggesting coordinated regulation within shared biological pathways. Notably, a perfect correlation (r = 1.0) was observed between *NPY2R* and 5-fluorouridine, indicating a strong regulatory link. Genes such as *IGF1*, *MAOB*, and *SAT1* correlated with multiple metabolites involved in amino acid and lipid metabolism. For instance, *IGF1* showed positive correlations with 5-oxoproline, L-glutamine, and LysoPC(16:1(9Z)/0:0), while *CD38* and *SAT1* were negatively correlated with stress- and lipid-related metabolites like jasmonic acid and mevalonic acid. Some metabolites, including LysoPC(16:1(9Z)/0:0) and jasmonic acid, emerged as central nodes, associating with several genes. Highly significant correlations (r > 0.97, *p* < 0.0001) between upregulated gene–metabolite pairs are shown in Figure 6B, and downregulated pairs are shown in Figure 6C. These results underscore key gene–metabolite interactions that may play critical roles in testicular metabolic regulation.

### 3.10. Molecular Docking and MDS Between Flavonoland and Target Proteins

**Figure 7:**
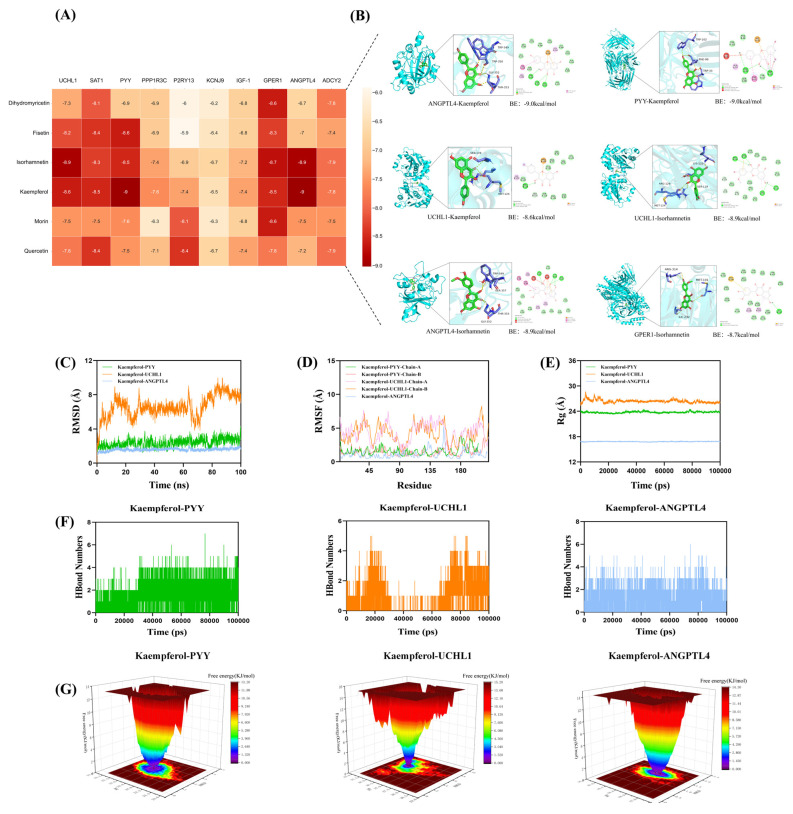
Interaction analysis between active compounds from fermented Chinese chive and target proteins involved in early testicular development. (**A**) Heatmap showing molecular docking binding energies between major flavonols and key target proteins. (**B**) Representative 2D and 3D molecular docking models of kaempferol with UCHL1, ANGPTL4, and PYY, and isorhamnetin with UCHL1, ANGPTL4, and GPER1. (**C**) RMSD (root mean square deviation) plots indicating the structural stability of protein–ligand complexes over time. (**D**) RMSF (root mean square fluctuation) plots reflecting residue-level flexibility within the complexes. (**E**) Rg (Radius of gyration) plots demonstrating the compactness of protein structures during simulation. (**F**) HBond (hydrogen bond) analysis showing the number of hydrogen bonds formed between ligands and proteins throughout the simulation period. (**G**) Gibbs free energy landscape illustrating the conformational stability and energy states of the complexes.

In molecular docking, a lower binding free energy reflects stronger ligand–protein interactions, indicating higher affinity and greater complex stability [51]. Major flavonols from fermented Chinese chive showed binding energies below −5.0 kcal/mol with proteins regulating early testicular development. Notably, kaempferol and isorhamnetin exhibited the strongest affinities (−9.0 kcal/mol) with *PYY*, *ANGPTL4*, and *UCHL1* (Figure 7A,B). Molecular dynamics simulations confirmed the stability of these complexes, supported by consistent hydrogen bonding interactions (Figure 7C–G). These results highlight the potential of chive-derived flavonols to modulate testicular development at the molecular level.

### 3.11. DEG qRT-PCR

**Figure 8:**
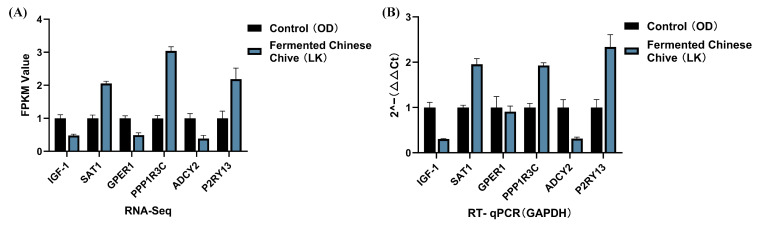
Validation of gene expression related to early testicular development. (**A**) Transcript abundance measured by RNA-seq for selected target genes. (**B**) Relative mRNA expression levels determined by RT-qPCR, normalized to *GAPDH*.

We randomly selected the six genes (*IGF-1*, *SAT1*, *GPER1*, *PPP1R3C*, *ADCY13*, and *P2RY13*) for RT-qPCR validation. The expression trends observed by qPCR were highly consistent with the transcriptome sequencing data, strongly confirming the high accuracy, reliability, and reproducibility of the transcriptome sequencing results (Figure 8A,B).

## 4. Discussion

This study shows that supplementing piglets with fermented Chinese chive modulates key pathways involved in metabolism, immunity, oxidative stress resistance, and testicular development. While no visible morphological differences were found between the supplemented (LK) and control (OD) groups at 28 days, transcriptomic and metabolomic analyses indicate significant functional improvements in the LK group that may precede structural changes. Previous research suggests that *A. tuberosum* extracts reduce ROS and protect mitochondrial integrity in testes, supporting the idea that fermented Chinese chive may enhance antioxidant defenses and create a healthier environment for early germ cell development [52].

### 4.1. Immune and Erythropoiesis-Related Hub Genes in the LK Group

PPI network analysis identified key upregulated hub genes in the LK group (*HP*, *LTF*, *SLC4A1*, *S100A12*, *S100A8*, *ALAS2*, *HBB*, *AHSP*, *KLF1*, *MMP9*, *RETN*, and *PGLYRP1*) linked to immunity, erythropoiesis, and inflammation. This suggests that fermented Chinese chive supplementation may strengthen host defense, support red blood cell function, and promote tissue remodeling. Haptoglobin (Hp) is a Sertoli- and germ cell-derived protein involved in testicular iron regulation and spermatogenesis [53]. Upregulation in the LFP group suggests a role in redox balance and testicular development. Lactoferrin mitigated Deoxynivalenol-induced damage by restoring spermatogenesis, preserving the blood–testis barrier, and reversing oxidative stress and inflammation, highlighting its potential to protect male fertility from mycotoxin toxicity [54]. During weaning, a proper pH balance—maintained by HCO_3_^−^/H^+^ transporters and ion channels—is vital for Sertoli cell function, blood–testis barrier formation, and early spermatogenesis. Hormonal control of these regulators supports healthy testicular development and future fertility [55]. *S100A8*, *S100A9*, and *S100A12*, crucial at the maternal–conceptus interface, may similarly support testicular development by modulating local immune responses and epithelial cell function. Their potential role in establishing immune privilege and cellular communication could be vital for early testis maturation and germ cell development [56]. *ALAS1*, known for its role in ovarian folliculogenesis, is hypothesized to similarly influence early testicular development by supporting mitochondrial function, steroidogenesis, and cell differentiation critical for testis maturation and male fertility [57]. A previous study reported downregulation of hbb post-spermiogenesis, suggesting that its role is limited to early testicular stages. In contrast, our observation of hbb upregulation during early testis development implies a possible role in oxygen regulation and redox balance [58]. Our identification of *KLF1* as a hub gene aligns with findings in chickens showing conserved roles of the *KLF* family in regulating cell proliferation, metabolism, and tissue development. This supports the idea that fermented Chinese chive modulates conserved KLF pathways to enhance early testicular and metabolic function [59]. Metalloproteinases regulate the testicular extracellular matrix, influencing sperm development and reproductive function, but their precise mechanisms remain unclear. This review highlights their physiological and pathological roles to better understand male fertility [60]. Our findings suggest that fermented Chinese chive (LK) may influence early testicular development in pigs by modulating the expression of immune-related genes such as *RETN*, potentially affecting testicular microenvironment stability and germ cell maturation [61]. *THSD7B* is known to regulate actin cytoskeleton reorganization, protein glycosylation, and cell adhesion, and is linked to angiogenesis. Its positive correlation with immune cells such as mast cells, B cells, CD8^+^ T cells, and NK cells suggests that it promotes immune activation. In cancer, *THSD7B* overexpression suppresses cell proliferation, viability, migration, and invasion. Thus, its downregulation in the OD group may affect cell adhesion and alter the neuronal microenvironment, despite its better-characterized roles in cancer and immunity [62]. Meanwhile, *TOX3* (TOX High Mobility Group Box Family Member 3) is a transcription factor predominantly expressed in the developing mouse brain, peaking at embryonic day E14 [63]. It co-localizes with neural stem and progenitor markers Nestin and Sox2 in radial glia and intermediate progenitors [63]. *TOX3* is a nuclear protein containing a high mobility group (HMG) box domain, which regulates calcium-dependent transcription in neurons through interaction with the cAMP-response-element-binding protein (CREB) [64]. Knockdown of *TOX3* in mouse embryos leads to reduced Nestin expression, decreased neural progenitor proliferation, and impaired migration to the cortical plate, indicating its crucial role in nervous system development. *TOX3* also functions as a neuronal survival factor, protecting neuronal cells from cell death by inducing anti-apoptotic transcripts and repressing pro-apoptotic ones [63]. Downregulation of *TOX3* in the OD group suggests reduced neural progenitor function and neuronal survival. The *CSMD1* in the OD group supports its role in protecting gonadal tissue, while its lower levels in the LK group imply that fermented Chinese chive may reduce the need for *CSMD1*-mediated complement regulation [65]. These results are aligned with our observation of an enhanced immune response and extracellular matrix remodeling genes in the LK group, suggesting that the fermented Chinese chive may activate alternative protective pathways to maintain testicular health.

### 4.2. Integration of Transcriptomic and Metabolomic Findings

Combined analysis revealed overlapping pathways—ferroptosis, insulin resistance, MAPK, Ras, Rap1, cGMP-PKG, and cAMP—highlighting the broad impact of fermented Chinese chive on testicular metabolism, stress response, and signaling [66]. Genes linked to metabolism (*PPP1R3C*, *TDH*, *PYGM*, *ARG1*, *ANGPTL4*) and immunity (*CD38*, *CXCR2*, *CSF3R*, *PYY*) were upregulated in the LK group. *PPP1R3C* enhances glycogen storage, providing vital energy for testicular cell growth and differentiation [67]. Adequate energy supply via glycogen metabolism supports Sertoli and germ cell proliferation, essential for testicular growth at early stages [67,68]. *PYGM,* primarily studied in glycogen metabolism, is also important in testicular cells, especially during periods of high energy demand such as cell proliferation and spermatogenesis [69]. *ARG1* expression in testicular tissue may support the high proliferative activity of Sertoli and germ cells during early development by regulating nitrogen metabolism and polyamine synthesis [70]. *ANGPTL4* (angiopoietin-like 4) regulates lipid metabolism by inhibiting lipoprotein lipase (LPL), affecting triglyceride clearance and fat deposition [71]. *ANGPTL4* is widely expressed in pigs and supports lipid metabolism and vascular remodeling. By regulating lipid availability for membrane synthesis and energy, and aiding vascular development, it may promote growth and functional maturation of the testis during early weaning [66]. *CD38* encodes a multifunctional enzyme involved in NAD^+^ metabolism and calcium signaling, both vital for cellular processes including proliferation and differentiation [72]. Calcium signaling, modulated by *CD38* activity, is essential for germ cell maturation and Sertoli–germ cell communication. The *CD38*-like molecule cloned from Nile tilapia is surface-expressed on B cells and is upregulated during B cell activation and differentiation, indicating its role in T cell-dependent immune responses [73]. Neutrophil recruitment during early inflammation depends on perivascular mast cells to trigger *CXCL1*/*CXCL2* release and on macrophages to maintain chemokine production for deeper tissue infiltration. Together, they coordinate an effective neutrophil response to infection or injury [74]. These findings support our hypothesis that fermented Chinese chive enhances metabolic regulation and immune responses, promoting healthy testicular development in piglets during early growth.

Fermented Chinese chive demonstrates multifaceted biological activities relevant to male reproductive health and neurofunction. Crucially, it upregulates genes critical for combating oxidative stress (*TFRC*, *PMAP-36*), cellular signaling and testis structure (*COL6A6*, *FOXA2*, *SAT1*, *RIN1*, *GRIA1*, *KCNJ9*), steroidogenesis and hormone signaling (*CYP11A1*), and neurotransmission (*NPY2R*, *P2RY13*, *GRPR*, *CPLX2*). This coordinated modulation suggests a potential role in enhancing testicular development and function. Male germ cells exhibit intrinsic susceptibility to oxidative damage due to cytoplasmic antioxidant deficits and peroxidation-susceptible polyunsaturated fatty acid-enriched membranes [7,75]. While physiological ROS generation regulates spermatogenesis [7], pathological oxidative stress impairs it via DNA damage and apoptosis [75]. The upregulation of *TFRC* is particularly significant within the context of male germ cell vulnerability. *TFRC*, essential for iron uptake and cellular response to oxidative stress, represents a key compensatory mechanism; its upregulation facilitates cellular iron import and potentially influences redox balance [76,77]. Thus, fermented Chinese chive contributes to strengthening cellular antioxidant defenses, particularly under metabolic or environmental stress. Concurrently, it elevates testosterone-associated metabolites by upregulating steroidogenic pathways and optimizes redox homeostasis through flavonoid-mediated ROS scavenging and metal chelation [16,17,78,79]. Although systematic evaluation of antioxidant supplements for male fertility is warranted, whole-food matrices like fermented Chinese chive offer enhanced safety through multicomponent synergy [80], analogous to the protective effects observed with the Mediterranean diet against environmental pollutant-induced infertility [81,82,83,84]. This integrated efficacy, potentially superior to isolated compounds due to adaptability to developmental plasticity [84,85,86], requires validation of dose–response dynamics for key bioactives. Furthermore, fermented Chinese chive supports testicular development by enhancing Gastrin-releasing GRPReins and spermatogenic pathways, evidenced by the upregulation of *FOXA2* (transcription), *SAT1* (polyamine metabolism), *RIN1* (signaling), *GRIA1* (synaptic transmission), and *KCNJ9* (ion channel function) [87,88,89]. The induction of the cathelicidin antimicrobial peptide *PMAP-36* adds an immunomodulatory dimension to its protective profile [90,91]. The upregulatory trend in *CYP11A1*—a key rate-limiting enzyme in steroidogenesis—suggests a potential enhancement in testicular steroidogenic capacity following fermented chive intervention. This may contribute to the improvement in testicular development, possibly through promoting gonadal maturation via initiation of steroid hormone biosynthesis [92]. *NPY2R*: Neuropeptide Y receptor involved in appetite regulation, stress response, and emotional behavior [93]. The gastrin-releasing (*GRPR*) peptide reduces hippocampal CA1 neuron excitability by enhancing inhibitory synaptic transmission, offering insight into its role in improving cognition and treating CNS disorders [94]. *CPLX2* is a potential tumor suppressor and prognostic biomarker for glioma by modulating hypoxia and inflammation pathways [95]. The regulation of these neurotransmission-related genes by fermented Chinese chive indicates a potential influence on brain–gut axis communication, mood, and cognitive function. Modulation of these pathways may contribute to the neuroprotective and nootropic effects attributed to fermented foods, possibly through microbiota-derived metabolites and their impact on host gene expression [96,97].

Conversely, OD-upregulated genes were associated with inflammation and prostaglandin signaling (*PLA2G4F*, *PTGER3*), steroidogenesis and hormone signaling (*IGF1*, *GPER1*, *ADCY2*, *TACR3*), extracellular matrix organization (*COL4A3*, *COL4A4*), and oxidative metabolism (*MAOB*, *AGXT2*). Prostaglandin E2 (*PGE2*) signaling through its receptors, including *PTGER3*, modulates systemic inflammation by regulating innate lymphoid cells and IL-22 production, highlighting the anti-inflammatory role of prostaglandins in immune responses. The role of prostaglandin receptors, such as *PTGER3,* in inflammation and immune regulation is further supported by studies showing their involvement in inflammatory diseases and immune cell modulation [98]. *COL4A3* and *COL4A4* encode alpha chains of type IV collagen, a major structural component of basement membranes essential for tissue integrity, especially in the kidney and sensory organs. Mutations in these genes are linked to Alport syndrome and other basement membrane disorders [99]. Studies have shown that *COL4A3* is involved in extracellular matrix deposition, and its downregulation can promote cell migration and invasion, highlighting its role in tissue structure and disease progression [100]. *IGF1* (insulin-like growth factor 1) plays a central role in growth, metabolism, and hormone signaling. It interacts with estrogen and androgen receptors, influencing gene expression and cellular responses in various tissues, including reproductive organs [101]. Overall, results indicate that fermented Chinese chive has improved growth and reproductive potential during critical early-life stages in pigs.

This study establishes the first multi-omics evidence in suckling piglets that fermented Chinese chive orchestrates coordinated transcriptional–metabolic dynamic regulation during critical testicular development, revealing novel interactions between flavonoid-mediated antioxidant defenses and steroidogenic activation. Nevertheless, causal attribution necessitates functional validation through in vitro interrogation of candidate genes. Furthermore, while the dietary intervention (fermented Chinese chive supplementation) was implemented to influence piglets via lactation during the 0–7 day period when solid feed intake is absent, the absence of compositional analysis of the maternal milk precludes definitive attribution of the observed effects solely to the fermented chive components versus potential alterations in milk nutrients or other mammary-derived mediators. Additionally, the absence of proliferation and apoptosis indices for key testicular cells, along with unmeasured testicular kisspeptin and sex steroid hormone levels, limits our ability to comprehensively assess the dynamics of testicular activation and maturation, including whether the observed activation leads to early expansion, requiring subsequent compensatory apoptosis to mature the testicular niche. Despite these constraints, our findings provide a foundational framework for nutritional intervention in suckling piglet developmental programming. Future studies should prioritize mechanistic validation using ex vivo porcine germline cultures, quantitative profiling of maternal milk components (particularly bioactive metabolites derived from fermented chives) to establish intervention–dose–response relationships, as well as comprehensive quantitative analysis of testicular cellularity and dynamics, complemented by measurement of kisspeptin and steroid hormone levels to elucidate niche maturation. In addition, longitudinal tracking across weaning-to-puberty transitions is also essential to assess sustained reproductive competence.

## 5. Conclusions

This study demonstrates that dietary supplementation with 3‰ fermented Chinese chive from late gestation through lactation can positively influence early testicular development by modulating metabolic and immune-related pathways in Songliao Black piglets. Although no significant differences were observed in gross testicular morphology, the LK group exhibited higher weaning weights, improved serum lipid profiles, and elevated IgG levels, indicating enhanced growth performance and immune status. Transcriptomic and metabolomic analyses revealed that fermented Chinese chive supplementation upregulated genes involved in energy metabolism, antioxidant defense, immune modulation, neuroendocrine signaling, and testicular function. These findings suggest that fermented Chinese chive supports metabolic activity, reduces oxidative stress, and promotes immune homeostasis, potentially facilitating the early activation of reproductive pathways in developing testes. Overall, fermented Chinese chive holds promise as a functional feed additive to improve growth and reproductive potential during critical early-life stages in pigs.

## Figures and Tables

**Table 1:** The effects of fermented Chinese chive on the growth performance of suckling piglets.

Category	Control (OD)	Fermented Chinese Chive (LK)
Piglet birth weight (kg/head)	1.23 ± 0.21	1.31 ± 0.29
Litter birth weight of piglets (kg)	12.24 ± 2.29	13.57 ± 2.08
1-week survival rate (%)	93.33 ± 3.21	95.65 ± 4.22
28 d weaning weight (kg)	6.57 ± 1.12 ^b^	7.48 ± 1.74 ^a^
28 d litter weaning weight (kg)	62.97 ± 4.92 ^b^	72.48 ± 11.01 ^a^

Different lowercase letters in superscript indicate significant differences (*p* < 0.05); identical letters or no letters indicate no significant difference (*p* > 0.05).

**Table 2:** The effects of fermented Chinese chive on serum biochemical indicators of suckling piglets.

Category	Control (OD)	Fermented Chinese Chive (LK)
TP (g/L)	62.75 ± 6.25	58.25 ± 6.95
ALB (g/L)	31.20 ± 4.60	26.55 ± 1.45
GLUC (mmol/L)	6.25 ± 1.19	5.25 ± 0.22
TG (mmol/L)	1.10 ± 0.18 ^b^	2.07 ± 0.03 ^a^
TC (mmol/L)	2.56 ± 0.36 ^b^	3.65 ± 0.05 ^a^

Different lowercase letters in superscript indicate significant differences (*p* < 0.05); identical letters or no letters indicate no significant difference (*p* > 0.05).

**Table 3:** The effects of fermented Chinese chive on serum antioxidant indicators of suckling piglets.

Category	Control (OD)	Fermented Chinese Chive (LK)
T-AOC (U/mL)	0.40 ± 0.06	0.53 ± 0.08
CAT (U/mL)	4.87 ± 0.48	4.54 ± 0.99
GSH-PX (U/mL)	654.17 ± 42.69	664.13 ± 23.77
SOD (U/mL)	49.97 ± 3.31	46.34 ± 3.37

Different lowercase letters in superscript indicate significant differences (*p* < 0.05); identical letters or no letters indicate no significant difference (*p* > 0.05).

**Table 4:** The effects of fermented Chinese chive on serum immune indicators of suckling piglets.

Category	Control (OD)	Fermented Chinese Chive (LK)
IgA (μg/mL)	271.67 ± 11.70	289.33 ± 17.13
IgM (mg/mL)	3.87 ± 1.42	3.97 ± 0.12
IgG (mg/mL)	15.03 ± 0.2 ^b^	19.87 ± 0.69 ^a^

Different lowercase letters in superscript indicate significant differences (*p* < 0.05); identical letters or no letters indicate no significant difference (*p* > 0.05).

## Data Availability

The raw data has been uploaded to NCBI with Bioproject accession no: PRJNA1291915.

## Supplementary Materials

The following supporting information can be downloaded at: https://www.mdpi.com/article/10.3390/antiox14091056/s1, Table S1: Basic feed composition and nutritional levels of sows and suckling piglets; Table S2: q-RT-PCR primers used for validation; Table S3: Statistics summary of RNA-Seq data; Table S4: Comparison of RNA Seq data; Table S5: The KEGG pathways of differential gene expression; Table S6: The GO pathways of differential gene expression; Table S7: Untargeted metabolites detected in LC-MS/MS; Table S8: The KEGG pathways of differential metabolites expression; Table S9: Higher expression DEGs identified in Joint analysis with metabolites.
