# Supplementary material for: Integrated Transcriptomic and Metabolomic Analysis Reveals Regulatory Effects of Fermented Chinese Chive on Early Testicular Development in Piglets
Source: Antioxidants (Basel). 2025 Aug 28;14(9):1056. doi: 10.3390/antiox14091056 (PMC12466648; doi:10.3390/antiox14091056)
Supplement: Supplementary file 1 [file antioxidants-14-01056-s001.zip › Table S1.pdf]

Table S1. Basic feed composition and nutritional levels of sows and suckling piglets

Ingredients and nutrient levels of the basal diet fed to sows and suckling piglets (as-fed basis)

**Ingredients %**	**Category**	
	**Sow feed**	**Diets for sucking piglets**
Corn (Grade 1)		55.26
Corn (Grade 2)	43.07	
Puffed corn		10
Barley	3.6	
Wheat mixture	10	
Rice bran	6	
Soybean oil	2.5	1.3
Whey powder (low protein)		2.5
Soybean meal (≥ 43%)	18.6	
Soybean meal (≥ 45%)		10.3
Soybean Enzymatic Hydrolyzed Protein (EP30)		7
Expanded soybean		4
Fish meal Japanese grade		1.2
DDGS High Fat Level 1	4	
Rice bran meal	8.5	4
4.44% Conservation Premix [1)]		4.44
3.73% Premix for lactating sows[2)]	3.73	
Total	100	100
Nutrient level[3)]		
Crude protein	≥17.0	≥16.5
Crude fiber	≤5.0	≤8.0
Coarse ash content	≤9.0	≤12.0
Calcium	0.3-1.3	0.6-1.5
Sodium chloride	0.2-1.5	0.2-1.2
Lysine	≥1.35	≥1.1
Water content	≤14.5	≤14.5

Note:

[1]Each kilogram of the 4.44% nursery premix contains:

[2]Each kilogram of the 3.73% lactating sow premix contains:

[3]Nutrient levels were calculated values
